# Sleep-circadian modulation of autophagy and glymphatic function: failure of coordinated brain clearance in Parkinson’s disease

**DOI:** 10.1038/s41531-026-01427-3

**Published:** 2026-06-08

**Authors:** Shamaila Zafar, Jay S. Schneider

**Affiliations:** 1 Department of Physiology and Pharmacology, Isakson Center for Neurological Disease Research, College of Veterinary Medicine, University of Georgia, Athens, GA USA; 2 Department of Pathology and Genomic Medicine, Thomas Jefferson University, Philadelphia, PA USA

**Keywords:** Cell biology, Neurology, Neuroscience

## Abstract

Parkinson’s disease (PD) involves α-synuclein accumulation and progressive neurodegeneration. This review proposes that PD reflects failure of sleep-circadian regulated brain clearance network integrating intracellular autophagy and extracellular glymphatic transport across neurons, glia, and blood-brain-barrier. Disruption of sleep-architecture and circadian-timing desynchronizes these processes, impairing protein clearance and promoting pathology. This framework positions sleep-circadian dysfunction as an upstream mechanism, suggesting that restoring sleep-circadian regulation may improve protein clearance and support disease-modifying strategies.

## Introduction

Parkinson’s disease (PD) is an age-related, multifactorial, neurodegenerative disorder characterized by the progressive degeneration of dopaminergic neurons in substantia nigra pars compacta (SNpc) and the pathological accumulation of misfolded proteins, most notably alpha-synuclein (α-Syn)^1,2^. Under physiological conditions, α-Syn regulates synaptic vesicle dynamics^3,4^; however, post-translational modifications such as Ser129 phosphorylation promote α-Syn misfolding into oligomeric and fibrillar species that accumulate as Lewy pathology and contribute to neuronal dysfunction and loss^5,6^.

As the second most common neurodegenerative disease after Alzheimer’s disease (AD), PD affects millions of individuals worldwide and imposes a rapidly growing societal and clinical challenge^7–9^. While classical motor symptoms (bradykinesia, rigidity, tremor, postural instability, gait disturbances and difficulties with speech)^10^ arise from dopamine deficiency in the striatum and basal ganglia circuitry dysfunction^11^, non-motor features, including cognitive impairment, autonomic dysfunction, mood disturbances, and especially sleep disorders, are increasingly recognized as prominent features of the disease and attributed in part to dopaminergic dysfunction along with dysfunction and/or neurodegeneration in other neurochemical systems and in brain regions, including the cortex, brainstem, and limbic system^12–14^.

## Sleep-circadian physiology and its disruption in PD

**Fig. 1:**
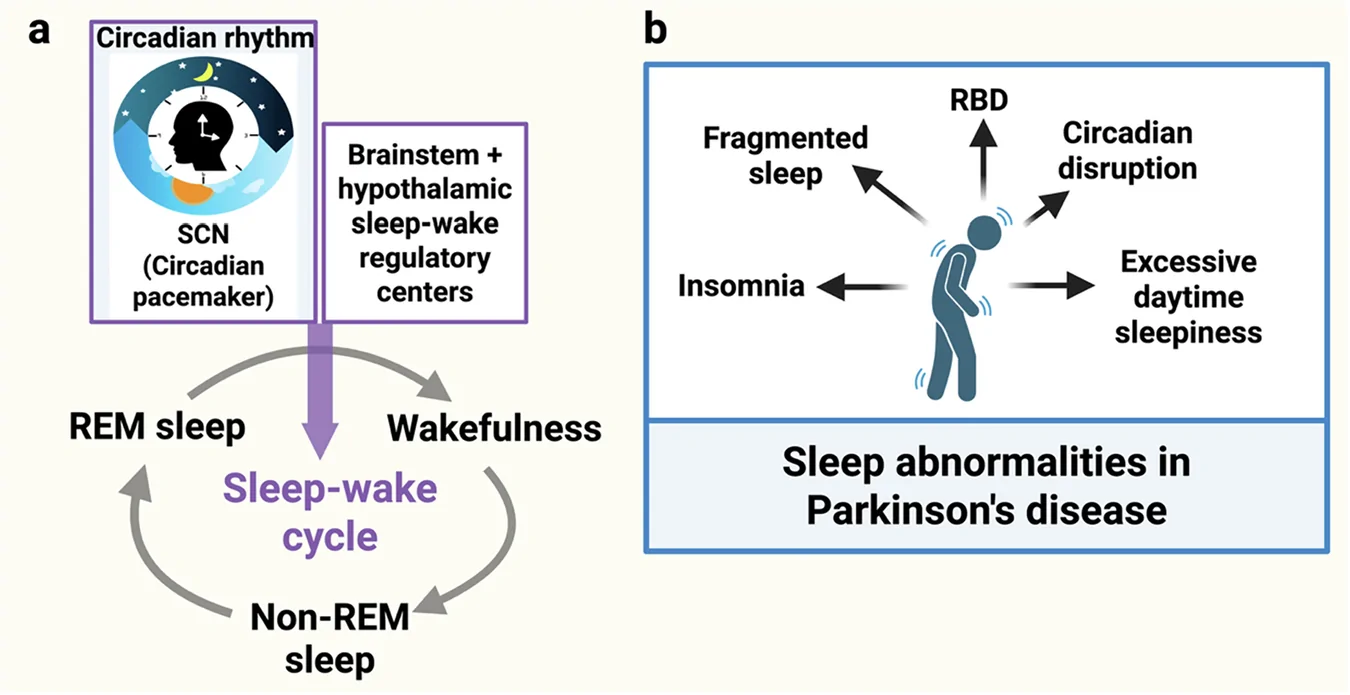
Sleep-wake regulation and sleep abnormalities in Parkinson’s disease. **a** Schematic representation of the sleep-wake regulatory system. The circadian pacemaker, suprachiasmatic nucleus (SCN) interacts with hypothalamic and brainstem sleep-wake regulatory centers to coordinate transitions between wakefulness, non-REM sleep, and REM sleep, thereby maintaining the organization of the sleep-wake cycle. **b** Major sleep disturbances observed in Parkinson’s disease, including insomnia, fragmented sleep, REM sleep behavior disorder (RBD), circadian disruption, and excessive daytime sleepiness, which collectively reflect disruption of normal sleep architecture and circadian regulation. REM= Rapid eye movement. (Created in https://BioRender.com).

Sleep-wake cycling refers to the dynamic alternation between wakefulness, non-rapid eye movement (non-REM), and REM sleep, each defined by distinct patterns of neuronal activity and coordinated shifts in key neuromodulatory systems, including norepinephrine (NE), serotonin, acetylcholine, orexin, and GABAergic signaling, which collectively regulate circuit excitability, metabolic demand, and processes critical for intra- and extra-cellular homeostasis. This cycle is temporally organized by the circadian system which comprises suprachiasmatic nucleus (SCN) as the central pacemaker and distributed network of other brain regions, including brainstem nuclei (locus coeruleus, dorsal raphe, pedunculopontine and laterodorsal tegmental nuclei), hypothalamic centers (orexinergic neurons of the lateral hypothalamus and the ventrolateral preoptic area), and thalamocortical circuits that collectively coordinate sleep-wake transitions and architecture to environmental light-dark cues and coordinates downstream neural and hormonal rhythms^15–17^ (Fig. 1). In PD, sleep abnormalities such as insomnia, REM sleep behavior disorder (RBD; a parasomnia characterized by loss of normal muscle relaxation during REM sleep, resulting in dream-enactment behaviors such as talking, shouting, or movements during sleep), circadian disruption, and excessive daytime sleepiness (Fig. 1) frequently emerge years, often decades, before the onset of motor dysfunctions^18–20^, which raises a critical question: are sleep abnormalities merely early clinical markers or do they actively participate in shaping the disease vulnerability?

Sleep may not simply be a passive state disrupted by neurodegeneration but rather a dynamic physiological process essential for maintaining neuronal homeostasis. Sleep disturbances likely arise due to disruption in the basal ganglia’s regulation of sleep-wake transitions due at least in part to SNpc dopaminergic neurodegeneration below the threshold for emergence of motor symptoms^21^. Additionally, impaired NE and serotonin-mediated control of REM-non-REM cycling^22,23^, loss of hypocretin (orexin)-producing neurons in the lateral hypothalamus projecting to monoaminergic/cholinergic nuclei^24^, cholinergic degeneration within REM regulating circuit of brain stem and basal forebrain^25,26^, and circadian pacemaker dysfunction in the SCN and downstream melatonin alterations^27^ are also involved. These early circuit-level disruptions suggest that sleep abnormalities in PD are not merely symptomatic but reflect fundamental alterations in neural systems governing physiological homeostasis. Such alterations can selectively destabilize the dynamic coordination of intracellular and extracellular clearance mechanisms.

## Parkinson’s disease pathology and brain clearance systems

Progressive dopaminergic (DA) neuron loss in the SNpc is the primary pathological characteristic of PD. The precise mechanisms that initiate and perpetuate this neuronal vulnerability remain incompletely understood. Mitochondrial dysfunction and oxidative stress have emerged as central, self-perpetuating drivers of neurodegeneration, leading to depletion of cellular energy stores, dysfunctional cellular signaling, impaired autophagy, and potentially contributing to glymphatic dysfunction^28^. Recent evidence suggests that the sleep-wake cycle may profoundly influence mitochondrial function and oxidative stress, where wakefulness acts as an oxidative challenge while sleep functions as a mito-restorative period, and in that way, may regulate neuronal bioenergetic homeostasis^29^.

Substantia nigra pars compacta DA neurons, due to their high basal metabolic rate of activity (related to fast action potential firing, pacemaking activity, and extensive arborizations), are particularly dependent on mitochondrial energy production and consequently are particularly vulnerable to mitochondrial dysfunction and oxidative stress^30^. Genetic mutations (e.g., *LRRK2, SNCA, PARK7 (DJ-1), PINK1, parkin, GBA1*), environmental toxicant exposures, and age-related cellular stress disrupt critical cellular pathways involved in mitochondrial quality control, leading to mitochondrial damage and excessive production of potentially harmful reactive oxygen species (ROS)^31–33^.

**Fig. 2:**
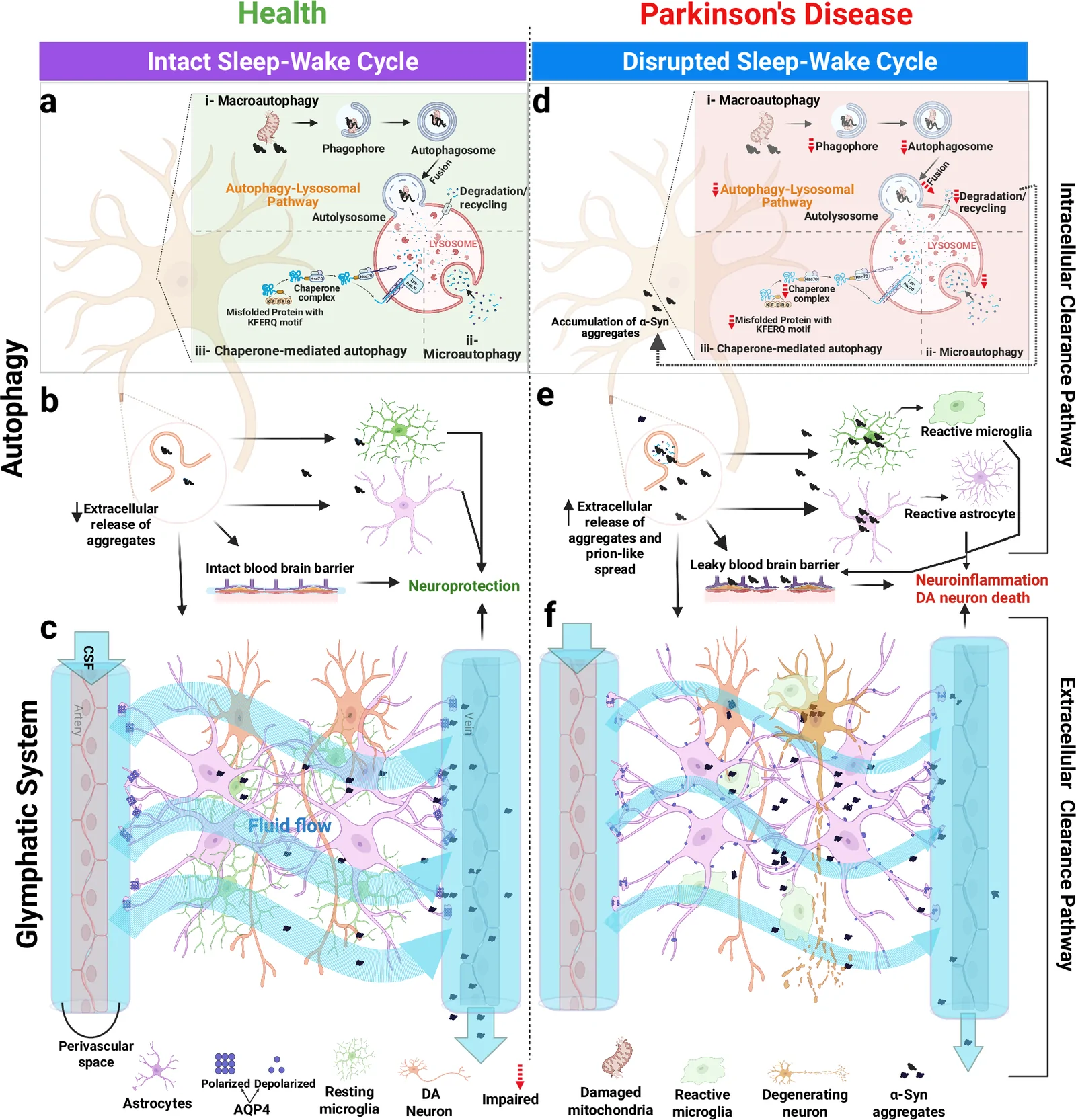
Clearance pathways in health and Parkinson’s disease. Under healthy condition with intact sleep-wake cycle, **a** Autophagic breakdown of intracellular wastes by i) macroautophagy (breakdown of damaged organelles and large protein aggregates through autophagy-lysosomal pathway)^45,155^, ii) chaperone mediated autophagy (engulfment of motif linked wastes proteins by lysosome)^156^, iii) microautophagy (endocytosis of small wastes by lysosomes)^157^; **b** Extracellular release of α-Syn aggregates^64^ and response of microglia^158^, astrocytes^70^ and blood brain barrier^96,97^ to take in and breakdown aggregates through autophagy and maintain homeostasis. **c** Glymphatic system characterized by flow of CSF in perivascular spaces facilitated by astrocytic expression of water channel AQP4 arrayed at their endfeet facing vessels (polarized), through the brain parenchyma and its exchange with fluid in interstitial spaces and removal of aggregates into perivenous spaces, with flow into the peripheral circulation^35^. In Parkinson’s disease with disrupted sleep-wake cycle, **d** dysfunctional autophagy results in impaired degradation and recycling of waste, causing increased pathogenic α-Syn aggregate burden inside cells^48,49^. This leads to **e** increased extracellular release of α-Syn and potentially prion-like spread^65,66^ as well as uptake by microglia^88^ and astrocytes^76^, converting them to their reactive phases, the latter potentially contributing to a leaky blood-brain barrier^100^ and creating a vicious cycle of inflammation leading to neurodegeneration. **f** Failure of glymphatic system to clear the aggregates, possibly due to mislocalization of polarized AQP4 from astrocytic endfeet to surfaces close to the astrocytic cell body (depolarization), resulting in the accumulation of more aggregates, exacerbating the neurodegeneration of dopaminergic neurons^128,129^. (Created in https://BioRender.com).

To counteract mitochondrial damage and maintain cellular homeostasis, neurons rely on tightly regulated intracellular and extracellular clearance systems. Autophagy represents a highly conserved intracellular degradative mechanism critical for eliminating misfolded proteins, damaged organelles, and toxic aggregates, thereby maintaining neuronal homeostasis^34^ (Fig. 2a). Complementing this is an extracellular brain-wide waste-clearance pathway, known as glymphatic system, that utilizes CSF to flush out extracellular waste products, including metabolic wastes and damaged proteins and organelles from the brain parenchyma^35^ (Fig. 2c). Together, these intracellular and extracellular systems form an integrated clearance network that maintains proteostatic balance.

Interestingly, both autophagic and glymphatic clearance pathways exhibit circadian modulation, with sleep emerging as a key physiological state that is increasingly recognized to modulate these processes. Experimental and clinical studies demonstrate that glymphatic transport is enhanced during sleep, particularly during slow-wave (non-REM) sleep, when the interstitial space expands, and CSF production is increased^36–38^. In parallel, autophagic activity exhibits circadian rhythmicity and is modulated by sleep-wake state transitions^39–41^. These observations suggest that clearance failure in PD may not reflect a uniform decline in the degradative capacity but rather a disruption in the dynamic coordination of clearance processes across sleep-wake cycles.

In this review, we synthesize evidence across autophagy and glymphatic function, and their regulation by sleep-wake cycle and circadian timing, to propose a unifying framework in which PD pathogenesis reflects a failure of dynamically coordinated brain clearance. By positioning sleep architecture and circadian timing as important regulators of clearance dynamics, we can reframe PD not only as a disorder of protein aggregation and its deficient clearance but also as a disease of disrupted sleep-state-dependent regulation of brain homeostasis, with implications for defining early biomarkers and for developing disease-modifying therapeutic strategies.

## Autophagy: a key intracellular clearance pathway under sleep-circadian regulation

Autophagy represents a primary intracellular pathway responsible for maintaining proteostasis and is potentially influenced by sleep circadian regulation. Under physiological conditions, autophagy operates at a basal but essential level to remove damaged organelles and misfolded proteins, a function particularly critical in post-mitotic neurons to preserve long-term sustainability^42^. This process involves sequestration of dysfunctional cytoplasmic components into double-membrane vesicles called autophagosomes, which then fuse with lysosomes for degradation and recycling (Fig. 2a).

Autophagic activity is tightly regulated by metabolic signaling pathways. Under nutrient-rich conditions, mTORC1 suppresses autophagy, whereas energy depletion activates AMPK-ULK1 signaling to initiate autophagosome formation^43^. Cargo selection is mediated by LC3 lipidation that binds adaptor and receptor proteins (p62, BNIP3, NIX, optinurin), enabling selective targeting of ubiquitinated protein aggregates and damaged mitochondria for degradation^44,45^. Efficient autophagy, therefore, depends on coordinated regulation of initiation, cargo recognition, vesicular trafficking, and lysosomal competence^46,47^.

Autophagic dysfunction is a well-established pathological hallmark of PD with evidence of impaired autophagosome maturation, vesicular trafficking, lysosome dysfunction, and reduced degradative capacity across neuronal and glial populations^48,49^. Chronic mitochondrial damage and oxidative stress place sustained demand on autophagy, resulting in pathological α-Syn accumulation that further exacerbates this process by impairing lysosomal integrity, and trafficking machinery. Genetic risk factors associated with regulatory genes including LRRK2, PINK1, Parkin, DJ-1, ATP13A2, and GBA1, converge on these pathways, collectively reducing degradative capacity and promoting accumulation of α-Syn aggregates^32,33,50–53^. Additional regulatory disruptions, such as impaired TFEB activation, ESCRT dysfunction, and reduced BAG3 expression, further constrain compensatory autophagic responses and weaken proteostatic resilience^54–56^.

Importantly, autophagy is not constitutively active but exhibits circadian rhythmicity and sleep-wake-dependent modulation. Early studies in rodents demonstrated that in peripheral tissues, autophagic activity follows a circadian rhythm, with the autophagic activity rising and falling across the day-night cycle^57^. Subsequent evidence extended this paradigm to the CNS, where both sleep and circadian processes interact with autophagic machinery. In the mouse hippocampus, autophagic markers (LC3 and beclin-1) displayed robust 24-hour rhythmic expression, with autophagosome associated LC3-II peaking at mid-light (resting) phase and reaching the lowest level at mid-dark (active) phase; however, chronic sleep fragmentation abolished this rhythmic LC3-II regulation^58^.

Experimental evidence further suggest that sleep-wake state differentially regulates distinct components of the autophagic pathway. Across genetic and pharmacological studies in *Drosophila*, wakefulness increases neuronal autophagosome abundance, whereas sleep (either spontaneous or drug-induced) suppresses autophagosome biogenesis without accelerating lysosomal degradation^59^, suggesting that sleep does not uniformly enhance autophagy but rather modulates the balance between autophagosome formation and clearance. Consistent with this, sleep induction in models of neurodegeneration, including *Drosophila* and rodent models of AD^39,60^ and rodent models of PD^61^, enhances autophagic flux and reduces pathogenic protein burden, whereas chronic sleep disruption induces maladaptive autophagic response and accelerates neurodegeneration.

These findings suggest that autophagy is dynamically gated across the sleep-wake cycle and sleep disruption, particularly fragmentation (disturbed slow-wave sleep) and circadian misalignment, may not simply reduce autophagic activity but instead disrupt its temporal coordination, leading to accumulation of autophagosomes, impaired flux, and inefficient clearance of α-Syn (Fig. 2).

Key experimental and clinical evidence linking sleep-circadian state to clearance processes is summarized in Table 1.

**Table 1:** Sleep-circadian regulation of clearance: key experimental and clinical evidence

Model/cohort	Sleep-circadian condition	Main finding	Study reference
Mouse (Liver)	Circadian rhythm/Day-night oscillation	Autophagy shows circadian rhythmicity across day-night cycle.	Ma et al.,^57^
Mouse (Brain)	Chronic sleep fragmentation	Autophagic markers, LC3 and beclin-1, show 24 h rhythm; sleep fragmentation abolishes rhythmic LC3-II regulation.	He et al.,^58^
Drosophila melanogaster (genetic sleep mutants; Autophagy gene manipulation)	Short sleep vs. sleep deprivation vs sleep promotion	Wakefulness increases autophagosome accumulation, sleep reduces it; altering autophagy levels in turn changes sleep behavior, demonstrating bidirectional regulation between sleep and autophagy.	Bedont et al.,^59^
Mouse (PD models; VMAT2- deficient and A53T mouse models)	Slow-wave sleep modulation	Slow-wave sleep influenced synucleinopathy and proteostatic processes	Morawska et al.,^61^
Patients with RBD/iRBD	REM sleep disruption (prodromal PD)	Elevated α-Syn species in neuron-derived extracellular vesicles, indicating altered extracellular α-Syn dynamics during early sleep disturbance states	Neilson et al.,^67^; Wang et al.^68^
Astrocyte- BMAL1 knockout mice	Circadian clock disruption	Impaired autophagy and lysosome function, reactive astrocyte phenotype.	McKee et al.,^81^
Mouse (in vivo imaging of astrocytes)	Sleep vs. wake state transitions	Astrocytes exhibit sleep-state-dependent Ca²⁺ signaling and transcriptional dynamics that regulate neuronal network activity and homeostasis, supporting a role as active modulators of brain-state-dependent clearance processes	Bojarskaite et al.,^80^
Mouse (MPTP-PD model)	Circadian clock disruption	Disruption of circadian clock component “REV-ERB-alpha” increased microglial activation and NLRP3 inflammasome signaling	Kou et al.^92^
Mouse, Human/ mouse BBB cell culture, Drosophila model	Active phase vs. Rest phase	Efflux activity increased during active phase and reduced during rest phase	Zhang et al.^105^; Zhang et al.^104^
Mouse model of sleep deprivation	Chronic sleep restriction/fragmentation	Increased BBB permeability, altered tight junction integrity, and neuroinflammatory changes, indicating that sleep loss compromised barrier integrity and clearance function	He et al.^106^ Sun et al.^107^
**Key glymphatic-relevant evidence**			
Mouse	Natural sleep vs. wake	Sleep increases interstitial space and enhances CSF-ISF exchange, improving waste clearance.	Xie et al.^114^
Mouse	Non-REM LC-NE oscillations	NE-driven vasomotion during Non-REM pumps CSF; disrupting these rhythms reduces glymphatic influx.	Hauglund et al.^115^
Mouse (AQP4 models)	Impact of AQP4 genetic deletion/mislocalization on natural sleep	AQP4 polarization supports sleep-linked glymphatic function; loss/mislocalization blunts it.	Mestre et al.^110^
Human	Slow-sleep wave oscillations	Coupled neural, vascular, and CSF oscillations consistent with sleep-driven CSF dynamics.	Fultz et al.^146^
Patients with PD + cognitive decline	Weakened global activity-CSF coupling	Reduced coupling suggests impaired sleep-driven CSF flow dynamics in PD cognitive decline.	Han et al.^145^
Patients with PD and RBD	RBD phenotype	Reduced glymphatic metrics in PD/RBD cohorts.	Si et al.^147^
Isolated RBD multi-cohort who later diagnosed with PD	iRBD phenoconversion	Lower glymphatic index in iRBD; supports prodromal clearance vulnerability marker.	Ayral et al.^144^
Patients with PD	Reduced slow-wave activity/RBD	Slow-wave sleep reduction and RBD correlate with increased PVS and worse motor and non-motor outcomes.	Meinhold et al.^148^
Patients with PD	AQP4 polymorphism linked to sleep disturbance	AQP4 variants associate with RBD and hallucinations more than overall PD risk, indicating sleep-stage-linked glymphatic vulnerability.	Agliardi et al.^149^
Mouse (intranasal oligomeric αSyn model)	Chronic circadian disturbance/sleep loss	Circadian disturbance increases nitrated α-Syn, microglial activation, DA neuron loss; sleep loss weakens glymphatic clearance and increases oxidative/inflammatory stress.	Zhang et al.^150^

*LC* locus coeruleus, *NE* norepinephrine, *REM* rapid eye movement, *RBD* REM-sleep behavior disorder, *iRBD* isolated REM sleep behavior disorder, *PVS* perivascular sace.

## Sleep and extracellular release of α-Syn: Interactive contributors to PD disease progression

When intracellular degradative capacity (autophagic clearance) becomes overwhelmed, neurons resort to non-classical secretory routes to excrete accumulated aggregates into extracellular spaces^62^. These routes include exosome-mediated release via extracellular vesicles^63^ and lysosomal exocytosis^64^, enabling extracellular α-Syn to be internalized by neighboring neurons, glial cells, and blood-brain-barrier (BBB)-forming cells, seeding further aggregation in them in a prion-like manner, promoting the spread of Lewy pathology across other brain regions^65,66^ (Fig. 2e).

**Fig. 3:**
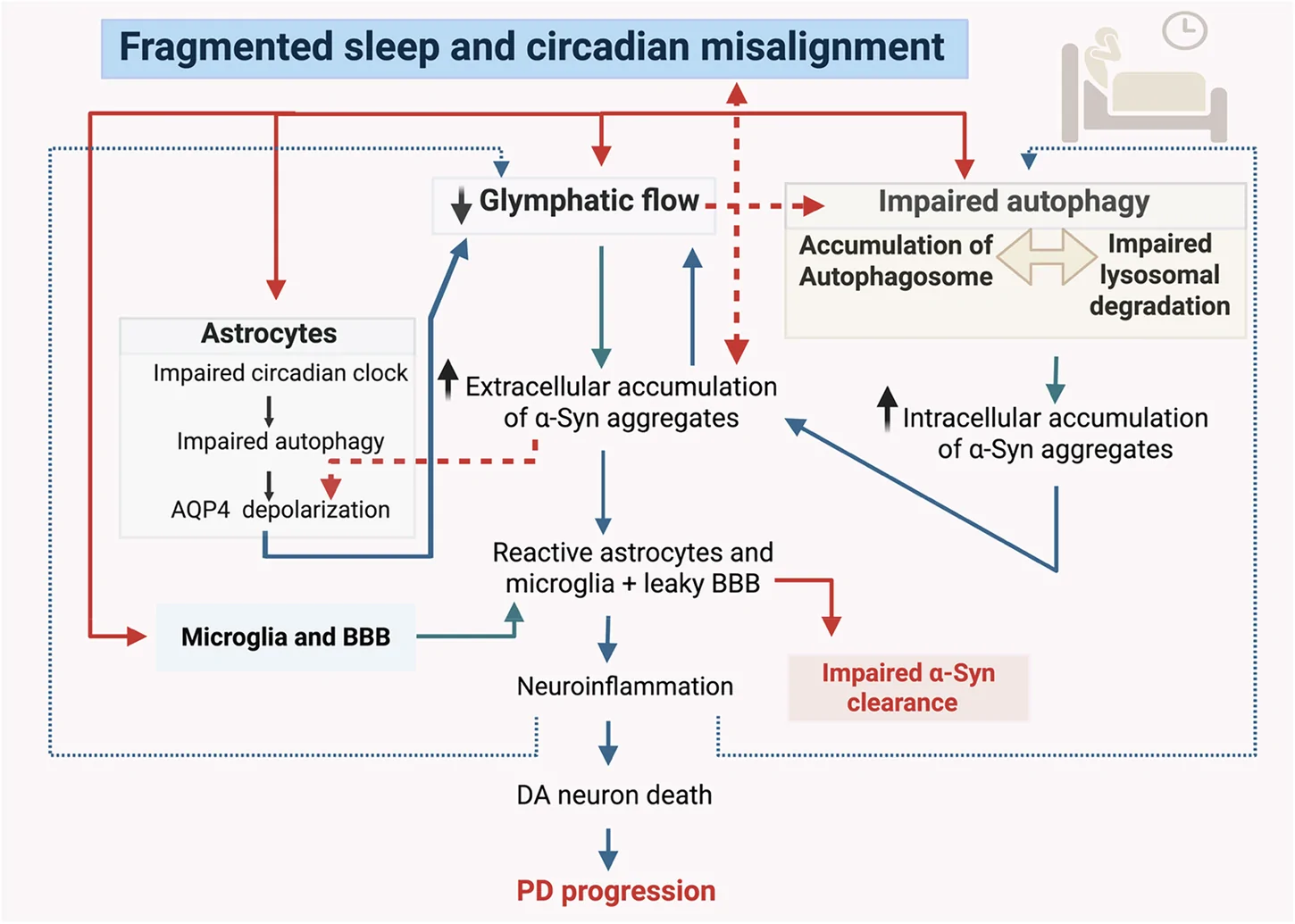
Conceptual model linking sleep fragmentation and circadian misalignment to coordinated clearance failure in Parkinson’s disease (PD). Sleep fragmentation and circadian misalignment are proposed to impair intracellular autophagy and extracellular glymphatic clearance through temporally desynchronized regulatory mechanisms. Impaired autophagy promotes intracellular α-synuclein (α-Syn) accumulation, while reduced glymphatic flow limits extracellular clearance, together increasing protein burden. Astrocytic dysfunction and AQP4 depolarization further compromise glymphatic transport. Accumulated α-Syn drives impaired glymphatic flow, glial reactivity, blood-brain barrier (BBB) disruption, and neuroinflammation, and may disrupt sleep-circadian regulation, reinforcing clearance failure and accelerating dopaminergic neuron loss and disease progression. Solid blue arrows represent relationships supported by experimental or clinical evidence; solid red arrows indicate hypothesized regulatory links supported by converging but incomplete evidence; dashed red arrows denote speculative/hypothetical relationships; blue dotted arrows indicate feedback loops. (Created in https://BioRender.com).

Sleep-wake state may critically influence the extracellular dynamics of α-Syn. Sleep disturbances such as RBD, particularly isolated RBD (iRBD), are strongly associated with prodromal synucleinopathies and early spread of α-Syn pathology across brain regions^67^. Patients with RBD/iRBD exhibit elevated levels of α-Syn species in circulating neuron-derived extracellular vesicles, indicating that extracellular α-Syn handling is altered early in prodromal PD^68,69^. These observations suggest that sleep-state disruption may not only coincide with but actively influence extracellular α-Syn dynamics. One possibility is that early α-Syn pathology within REM-regulating brainstem circuits contributes to both RBD and altered extracellular α-Syn profiles. However, whether sleep disruption directly drives these extracellular α-Syn changes or instead reflects early impairments in intracellular and extracellular clearance systems remains unclear (Fig. 3).

## Astrocytes as sleep-circadian integrators of autophagic clearance

Astrocytes exhibit intrinsic circadian regulation and occupy a strategic position at the interface of intracellular degradation and extracellular clearance, enabling coordinated control of proteostasis across system. Under physiological conditions, astrocytes limit the spread of extracellular α-Syn by actively taking it up through Toll-like receptor-mediated endocytosis and micropinocytosis and routing it to the autophagy-lysosomal system for degradation^70–74^. However, sustained uptake of pathological α-Syn species, particularly oligomers and pre-formed fibrils, rapidly overwhelm this system^75^. Chronic intracellular accumulation of α-Syn impairs autophagosome-lysosome fusion, reduces lysosomal degradative capacity, and induces mitochondrial and inflammatory stress, ultimately shifting astrocytes toward a reactive, autophagy-deficient, neurotoxic phenotype^76,77^. This neurotoxic astrocyte phenotype exhibits altered secretory profiles, including increased release of inflammatory cytokines and limited secretion of growth factors, which together compromise DA neuron integrity by exacerbating intraneuronal stress^73,76^. Autophagic impairment in astrocytes further disrupts the polarized localization of key perivascular component, Aquaporin-4 (AQP4), linking intracellular dysfunction to impaired glymphatic clearance and α-Syn accumulation^78,79^ (detailed mechanisms discussed in the later section). This establishes a mechanistic bridge between intracellular degradative failure and extracellular clearance deficits, amplifying proteostatic imbalance.

Emerging evidence suggests that astrocytic clearance capacity is tightly coupled to brain-state and intrinsic circadian regulation. Astrocytes exhibit sleep-state-dependent functional and transcriptional dynamics, mediated in part through intracellular Ca²⁺ signaling and gliotransmission, which regulate neuronal excitability and network synchronization across sleep-wake cycles^80^. These observations position the astrocytes as active regulators of brain-state transition rather than passive supportive cells. In another evidence, astrocyte-specific disruption of the core clock gene *BMAL1* has been shown to impair autophagic and lysosomal function and promote astrocytic reactivity^81^, suggesting that circadian integrity can directly modulate astrocytic clearance capacity.

In this context, astrocytes act as spatiotemporal integrators of brain clearance processes by coordinating autophagy, AQP4-dependent glymphatic transport, and intrinsic circadian and sleep-state dynamics. Given that astrocytic AQP4 polarization and metabolic functions are enhanced during consolidated sleep, disruption of sleep architecture and circadian timing in PD may impair astrocytic integrative role, leading to a breakdown of clearance coordination and promoting α-Syn accumulation.

## Microglia as sleep-state dependent regulators of clearance

Microglial function is dynamically modulated by sleep-wake state and circadian timing, positioning these cells as key mediators between neural activity states and extracellular proteostasis. Under physiological conditions, microglia efficiently internalize monomeric and aggregated α-Syn through receptor-mediated phagocytosis, routing internalized cargo to the autophagy-lysosomal pathway for degradation. Through intact autophagic flux, microglia restrict extracellular α-Syn spread, maintain interstitial homeostasis, and support neuronal survival^82,83^. However, this protective capacity is highly conditional. With aging or sustained exposure to pathogenic α-Syn species, microglial degradative pathways become overwhelmed, leading to impaired autophagosome formation, disrupted autophagosome-lysosome fusion, and lysosomal destabilization^84,85^. As autophagic clearance declines, microglia accumulate intracellular α-Syn and undergo a functional shift from containment of α-Syn accumulation to contributing to α-Syn propagation. Instead of fully degrading pathogenic cargo, autophagy-deficient microglia release α-Syn-containing extracellular vesicles that can seed aggregation in neighboring neurons and amplify extracellular α-Syn burden^86,87^. This transition is accompanied by NLRP3 inflammasome activation and inflammatory signaling, further amplifying neurotoxicity^88–90^.

Sleep and circadian mechanisms critically bias this functional state. In a study, sleep deprivation increases microglial activation and NLRP3 inflammasome signaling while sleep recovery reverses these effects^91^. In a PD model, circadian clock components such as REV-ERBα suppresses microglial NLRP3 signaling, while disruption of clock function enhances neuroinflammatory vulnerability^92^. In another study, BMAL1 deficiency alters microglial physiology and disrupts sleep-wake behavior, supporting the view that intrinsic clock programs shape microglial phenotype^93^. Microglia also participate in sleep regulation through P2Y12-Gi-coupled signaling, promoting sleep through intracellular Ca²⁺ activity and suppression of norepinephrine signaling, suggesting a bidirectional interaction between microglia and sleep-wake circuitry^94^. These findings suggest that sleep and circadian signals do not merely modulate microglial activity but may influence whether microglia operate in a degradative or propagative mode. Under intact sleep and circadian conditions, microglia favor efficient α-Syn clearance, whereas sleep fragmentation and circadian disruption may impair autophagic processing and promote inflammatory, vesicle-mediated α-Syn propagation.

## Blood-brain barrier as a circadian-gated clearance interface

The blood-brain barrier (BBB), formed by endothelial cells, astrocytes, and pericytes, serves as a regulated interface for the transport of extracellular α-Syn and is dynamically regulated by the sleep-circadian rhythms. Under physiological conditions, α-Syn crosses the BBB through regulated receptor-mediated mechanisms, including low-density lipoprotein receptor-related protein 1 (LRP1)^95^, with endothelial cells facilitating transcytosis, and pericytes contributing to uptake and autophagy-dependent degradation^96–99^. These coordinated processes likely buffer extracellular α-Syn. In PD, sustained exposure to pathological α-Syn disrupts the BBB integrity by activating pericytes and endothelial cells, promoting inflammatory signaling, disrupting tight junction integrity, and increasing vascular permeability^100–102^. As BBB-associated cells become autophagy-deficient, their ability to clear extracellular α-Syn declines, shifting the neurovascular unit from a protective interface to a contributor to pathology. Impaired pericyte degradation and endothelial dysfunction reduce brain-to-blood efflux of α-Syn, increase paracellular leak, and promote a pro-inflammatory vascular niche that facilitate further α-Syn accumulation and spread^100,103^.

Sleep and circadian rhythms dynamically modulate the BBB permeability and transport. Experimental studies demonstrate that BBB transport is under circadian control such that during the active/wake phase, efflux activity is elevated but is reduced during the rest/sleep phase, through clock-dependent mechanisms including intracellular magnesium regulation and gap-junction mediated intercellular signaling between barrier cell layers, thereby increasing BBB integrity^104,105^. Evidence also show that sleep disruptions impair BBB integrity. Chronic sleep restriction increases permeability, alters microvascular function, and reduces tight junction stability, while sleep fragmentation induces structural and functional deterioration of the barrier accompanied by neuroinflammatory changes^106–108^. These findings position the BBB as a sleep- and circadian-sensitive clearance interface. In PD, sleep fragmentation and circadian misalignment may reduce regulated α-Syn efflux while increasing vascular permeability and inflammatory signaling, thereby promoting extracellular α-Syn accumulation and propagation. Thus, BBB dysfunction represents not only structural breakdown but a temporally dysregulated component of impaired brain clearance.

Across neurons, glia, and BBB-forming cells, autophagy functions as a coordinated intracellular quality-control network that restrains proteotoxic stress and regulates α-Syn homeostasis in a sleep-wake-dependent manner. However, intracellular degradation alone cannot manage the substantial pool of α-Syn and waste proteins that accumulate in the extracellular space. Clearance of this burden depends on the glymphatic system, the brain’s specialized extracellular clearance mechanism. Understanding how the glymphatic system becomes compromised provides the essential next layer in understanding how protein burden accumulates and propagates in PD.

## Glymphatic system: a brain-wide clearance pathway

The glymphatic system is a brain-wide, glial-dependent clearance pathway that facilitates the removal of metabolic waste and neurotoxic proteins from the brain parenchyma^35^. Briefly, it is functionally analogous to the peripheral lymphatic system, which enables CSF to enter from the subarachnoid space (SAS) into periarterial perivascular spaces (PVS), penetrate the parenchyma where it mixes with interstitial fluid, and ultimately exit along perivenous routes before draining into meningeal lymphatic vessels^35^. Structurally, the system comprises the subarachnoid space, perivascular spaces, CSF, and astrocytic endfeet. Astrocytes form the glia limitans around the vasculature and are densely enriched with AQP4 water channels that are essential for driving convective CSF-interstitial fluid (ISF) exchange and promoting effective clearance of waste proteins^35,109,110^ (Fig. 2c).

Several physiological factors modulate glymphatic transport efficiency. Arterial pulsatility and vasomotion act as primary mechanical drivers of perivascular fluid movement in both the SAS and PVS^111,112^. Intracranial pressure (ICP) dynamics also influence flow, with elevated ICP increasing hydraulic resistance and impairing exchange^113^. Importantly, brain state profoundly alters glymphatic function, particularly during sleep when the interstitial space is expanded, and convective flux is enhanced, leading to increased clearance efficiency^114,115^.

## AQP4 polarization as a key regulator of glymphatic activity and α-Syn clearance

AQP4, due to its crucial role in regulating water homeostasis, is the primary focus of most research aimed at understanding glymphatic system function. It is highly enriched to perivascular astrocytic endfeet via interactions with dystroglycan-syntrophin complexes, where it provides low-resistance transmembrane water channels that couple vascular pulsatility to interstitial fluid movement^116–119^.

Converging evidence from genetic and pharmacological models, including AQP4 knockout mice and rats, α-Syntrophin (SNTA-1) knockout mice that exhibit abolished perivascular AQP4 polarization, and pharmacological blockade of AQP4 perivascular localization in mice using TGN-020, consistently show reduced CSF influx and slow clearance of extracellular solute, establishing perivascular AQP4 expression as essential for glymphatic function^110,120–123^. Interestingly, perivascular AQP4 localization (polarization), more than total expression, is the key determinant of clearance efficiency, especially for large macromolecules^120,124^.

Mislocalization of AQP4 from the perivascular endfeet of astrocytes can alter overall glymphatic activity, leading to impaired glymphatic transport, as may occur in neurodegenerative diseases like PD where AQP4 may be redistributed from astrocytic endfeet to the cell body, accompanied by reactive astrogliosis and reduced glymphatic clearance^78,123,125–127^. This implies that the integrity of astrocytic physiology in maintaining normal glymphatic function is important and plays a crucial role in clearing potentially pathological extracellular α-Syn (Fig. 2).

Emerging evidence shows that loss/downregulation of AQP4 aggravates α-Syn pathology and DA neurodegeneration, likely via impaired glymphatic efflux, whereas experimental inhibition of glymphatic function reduces clearance of misfolded α-Syn from brain parenchyma into CSF and exacerbates its propagation^128–131^. Consistent with this, intra-nigral α-Syn injections combined with pharmacological or genetic suppression of glymphatic flow (acetazolamide treatment, AQP4 deletion) increase parenchymal α-Syn retention, suggesting that the glymphatic system actively participates in handling extracellular α-Syn aggregate load^129,132^.

In A53T α-Syn overexpressing mice, surgical blockage of meningeal lymphatic vessels slowed clearance of a CSF tracer (Dextran-3) injected into cisterna magna, indicating impaired glymphatic transport. This intervention led to increased α-Syn accumulation, loss of perivascular AQP4 polarization, accelerated DA neuron loss in the SNpc, and worsening motor deficits, directly linking impaired glymphatic outflow to α-Syn-driven neurodegeneration^133^. Additionally, bilateral intrastriatal injection of α-Syn preformed fibrils (PFFs) in AQP4-heterozygous (AQP4 + /-) mice with reduced AQP4 expression, showed reduced glymphatic efflux, increased α-Syn aggregation, and increased SNpc neuron loss, compared to wildtype controls similarly exposed to PFFs, demonstrating that AQP4-dependent perivascular flow modifies α-Syn pathology progression^128^. In an earlier study, AQP4-knockout mice, when administered the neurotoxin MPTP, showed increased microgliosis and loss of SNpc DA neurons compared to wild-type controls similarly exposed to MPTP^134^. More recently, in LRRK2-R1441G mice, pathogenic LRRK2 phosphorylated and depolarized AQP4, impaired IFN-γ clearance via the glymphatic route and thus amplified neuroinflammation and dopaminergic neurodegeneration, demonstrating a link between a major genetic cause of PD to AQP4-mediated glymphatic failure^135^. Furthermore, molecular pathways, such as the MMP9/β-dystroglycan (β-DG) axis, regulate AQP4 localization^136^. Si et al.^137^ demonstrated in both MPTP-induced and A53T α-Syn mouse models of PD that MMP9-mediated cleavage of β-DG led to AQP4 depolarization, glymphatic dysfunction, and exacerbated dopaminergic neuronal loss^137^. However, it remains unclear whether extracellular α-Syn directly alters astrocytic AQP4 polarization or indirectly affects it by changing astrocyte behavior, raising the possibility of a feedback loop in which impaired glymphatic clearance both promotes and is further amplified by α-Syn accumulation (Fig. 3).

In short, following extracellular release, an intact and perivascularly polarized AQP4-glymphatic axis enables α-Syn clearance from the brain parenchyma into CSF. With this framework established, emerging clinical imaging studies consistently demonstrate that glymphatic flow is impaired in PD.

## Clinical evidence for glymphatic system impairment in PD

In vivo imaging studies in patients with PD have now provided evidence of dysfunctional glymphatic flow in the PD brain. Diffusion Tensor Imaging Analysis Along the Perivascular Space (DTI-ALPS) is used to measure the ALPS index, which is an MRI proxy of perivascular glymphatic transport. Evidence collected in different clinical settings in patients with PD compared with controls have shown a significantly reduced ALPS index and greater regional EPVS (Enlarged Perivascular Space) burden, especially in the basal ganglia and temporal lobe, and their positive correlation with more severe small-vessel disease, greater cortical atrophy, and worse cognitive and neuropsychiatric symptoms, as well as higher striatal dopaminergic loss as measured by DAT-SPECT (Dopamine Transporter Single-Photon Emission Computed Tomography) scans, greater motor symptom severity, and poor levodopa responsiveness^138–141^, supporting the view that impaired perivascular fluid handling is clinically relevant in PD rather than merely an incidental imaging feature. In longitudinal imaging studies, the lower baseline ALPS indices that progressively reduce over time and their association with subsequent cognitive decline in patients with PD suggest a clearance-specific contribution to cognitive vulnerability^142^.

Importantly, similar reductions in ALPS index are detectable in individuals at risk for PD, including those with isolated RBD (iRBD) and non-manifesting carriers of LRRK2 and GBA pathogenic variants, where lower baseline ALPS values are associated with an increased likelihood of later phenoconversion to clinically manifest PD^143,144^. Resting-state fMRI analyses further revealed reduced coupling between global neuronal activity and CSF dynamics in patients with PD and mild cognitive impairment, compared with cognitively intact patients and healthy controls. This decoupling, measured as diminished synchronization between global BOLD signal fluctuations and CSF flow, was associated with poorer cognitive performance and regional cortical vulnerability, while showing limited association with motor symptom severity^145^.

This evidence suggests that glymphatic clearance is compromised not only in established PD but also during prodromal stages and may reflect a disrupted temporal coordination between neural activity and CSF exchange, pointing to sleep-circadian regulation as a critical upstream modulator of glymphatic clearance efficiency and provides a mechanistic bridge to the sleep- and circadian-dependent control of brain clearance.

## Sleep and circadian rhythms as regulators of glymphatic system function in PD

A mechanistic link between sleep and glymphatic clearance was first demonstrated by in vivo two-photon imaging studies showing that natural sleep increases interstitial space volume by ~60%, markedly enhancing CSF-ISF exchange and accelerating the removal of waste proteins^114^. Subsequent work revealed that slow-wave sleep synchronizes neuronal down-states with large, low-frequency vascular pulsations that mechanically drive perivascular CSF flow^146^. More recent work identified infraslow oscillations of NE released from the locus coeruleus (LC) during non-REM sleep as key coordinator of slow vasomotion, producing rhythmic vascular dilations that actively pump CSF along perivascular pathways; disruption of these LC-NE rhythms, such as with sedative hypnotics, markedly reduces glymphatic influx^115^. In parallel, AQP4 polarization at astrocytic endfeet is maximized during consolidated sleep, whereas genetic deletion or mislocalization of AQP4 blunts sleep-dependent glymphatic activity^110^. Collectively, these mechanistic studies establish sleep as an active biomechanical and cellular state that enables efficient brain-wide clearance.

Human imaging studies reinforce the clinical relevance of this framework in PD. Weak coupling between global brain activity and CSF flow, in patients with PD and with cognitive impairment^145^ as well as DTI-ALPS imaging consistently demonstrating reduced glymphatic metrics in patients with PD and RBD^147^ and also in those with iRBD who were later diagnosed with PD^144^, position sleep-linked glymphatic dysfunction as a PD-specific prodromal vulnerability marker. Polysomnography-MRI-based analyses in patients with PD, further show that reduced slow-wave sleep activity and presence of RBD correlate with larger PVS counts, worse motor and non-motor outcomes^148^, while a recent genetic evidence in patients with PD demonstrates that AQP4 polymorphisms are selectively associated with RBD and hallucinations but not overall PD risks, collectively indicating that sleep-stage dependent glymphatic dysregulation preferentially contribute to non-motor symptoms expression in PD^149^.

Experimental models show a bidirectional link between sleep disruption and α-Syn pathology. In an intranasal oligomeric α-Syn mouse model, chronic circadian disturbance markedly increased nitrated-α-Syn accumulation, microglial activation, and dopaminergic neuron loss, while sleep loss simultaneously weakened glymphatic clearance and elevated oxidative and inflammatory stress, creating conditions that favor α-Syn misfolding and spread^150^. Accumulating α-Syn, in turn, disrupts brainstem and hypothalamic sleep-regulatory circuits, establishing a self-reinforcing loop in which impaired sleep accelerates proteotoxic burden, and proteotoxic stress further destabilizes sleep architecture^150^.

Together, these findings suggest that sleep and circadian rhythms do not merely modulate individual clearance pathways but act as dynamic organizers of an integrated clearance network, synchronizing astrocyte-mediated glymphatic transport with intracellular autophagic capacity (Table 1). In PD, early disruption of this dynamic coordination may decouple extracellular and intracellular clearance processes, lowering the brain’s resilience to toxic proteins’ stress and facilitating α-Syn accumulation and spread (Fig. 3). This framework raises a critical, forward-looking question: *could restoring circadian alignment and sleep architecture re-synchronize clearance systems before irreversible neurodegeneration ensues, thereby shifting sleep from a symptomatic marker to a disease-modifying target in PD?*

## Implications and future directions

Parkinson’s disease emerges not from failure of a single physiological pathway, but from a wide variety of potentially pathogenic processes^2^, including the progressive collapse of coordinated intracellular and extracellular clearance systems that depend on intact sleep-circadian regulation. The evidence synthesized in this review supports a framework in which autophagic degradation, extracellular α-Syn propagation, glymphatic transport, and astrocytic AQP4-dependent perivascular coupling form an integrated clearance network that is dynamically gated across the sleep-wake cycle. Disruption of this coordination, rather than uniform loss of degradative capacity, may represent a critical and underappreciated driver of PD pathogenesis.

An important implication of this model is that PD can be conceptualized not only as a proteinopathy and clearance-deficient state, but as a disorder of sleep-state-dependent clearance failure. Experimental and clinical data increasingly indicate that glymphatic transport and autophagic flux are maximized during specific sleep stages and circadian windows, particularly consolidated non-REM sleep^114,115,144,146,148,150^. Early disturbances in sleep architecture, circadian rhythmicity, sleep-state-dependent shift in microglial phenotype, or astrocytic clock integrity may therefore selectively impair clearance during these critical phases, which may further facilitate extracellular α-Syn propagation through vesicle-mediated spread. Importantly, this framework provides a mechanistic explanation for why sleep disorders such as iRBD and circadian disruption precede motor symptoms and predict phenoconversion to PD.

This perspective generates testable and translationally relevant hypotheses. First, glymphatic and autophagic dysfunction may emerge earliest during specific sleep phases, rather than as static deficits, suggesting that time-resolved biomarkers, such as sleep-phase-linked imaging metrics, CSF dynamics, or circadian oscillations in clearance-related markers, as well as in extracellular vesicles-based markers, could improve early detection of PD vulnerability. Second, astrocytes, microglia, and BBB may act as critical temporal integrators of clearance, linking circadian gene programs, autophagic capacity, AQP4 polarization, and perivascular fluid exchange. Disruption of astrocytic circadian integrity, as exemplified by BMAL1-dependent autophagic dysfunction, sleep-circadian disruption-mediated shift in microglial function from degradative to propagative inflammatory state, and fragmented sleep-induced disruption in BBB integrity, may therefore represent core points at which intracellular and extracellular clearance failure converge (Fig. 3).

From a therapeutic standpoint, this framework draws attention away from targeting individual clearance pathways in isolation and toward strategies that target multiple pathways that underlie PD pathophysiology as well as strategies that restore their temporal coordination. Interventions that stabilize sleep architecture, reinforce circadian rhythmicity, and/or preserve astrocytic perivascular coupling have the potential to simultaneously enhance glymphatic transport and autophagic efficiency^151–153^. Emerging preclinical evidence showing that sleep-targeted interventions restore AQP4 polarization, improve clearance dynamics, and attenuate neurodegeneration supports the feasibility of this integrated approach^154^. Importantly, such strategies may be most effective during prodromal stages of PD, when clearance networks remain structurally intact but are temporally dysregulated. Together, these findings position sleep and circadian regulation not merely as symptomatic targets, but as modulators of clearance network resilience with disease-modifying potential.

Thus, sleep disruptions in PD can be reframed from being a secondary symptom to an active driver of protein clearance failure (Fig. 3). By positioning sleep-circadian regulation as an upstream control of brain clearance, this framework offers a unifying explanation for early vulnerability, clinical heterogeneity, and disease progression. Future studies that integrate sleep physiology, circadian biology, and clearance dynamics across disease stages will be essential to test this model and to identify novel opportunities for early intervention and disease modification in PD.

## Data Availability

No datasets were generated or analyzed during the current study.
